# DICOM attribute manipulation tool: Easily change frame of reference, series instance, and SOP instance UID

**DOI:** 10.1002/acm2.70104

**Published:** 2025-04-09

**Authors:** Brian M. Anderson, Casey Bojechko

**Affiliations:** ^1^ Department of Radiation Oncology University of North Carolina Chapel Hill USA; ^2^ Department of Radiation Medicine and Applied Sciences University of California San Diego San Diego California USA

**Keywords:** DICOM

## Abstract

**Purpose:**

In radiation oncology, the integration and registration of multiple imaging modalities is a crucial aspect of the diagnosis and treatment planning process. These images are often inherently registered, a useful feature in most cases, but possibly a hindrance when registration modifications are required. To break this registration requires expert knowledge of file structure or specialized software, posing challenges, and potential errors in accidentally or unnecessarily changing other attributes. Barring these changes, the clinic would have to make do with imprecise registrations which compound overall treatment uncertainty.

To address these issues, we present a novel tool designed to simplify the task of changing three often edited attributes: the frame of reference, the series instance unique identifier (UID), and the SOP instance UID. The tool features an intuitive user interface that empowers practitioners, regardless of their expertise, to effortlessly modify these three commonly edited values.

**Validation methods:**

Publicly available brain MRI and TCI lung 4DCT images were used to evaluate the software. The ability to change the frame of reference, series instance identifier, and SOP instance identifier using the program was evaluated with both the RayStation treatment planning system and MIM.

**Software format and usage notes:**

The program is written in C#, easily distributed via GitHub and is compatible with any Windows computer with .NET 4.8 (the standard as of 2023).

**Potential applications:**

This innovation holds promise for improving the overall workflow efficiency and safety within radiation oncology and radiology, where breaking the frame of reference or changing the series/SOP UIDs is a common occurrence.

## 1 INTRODUCTION

The Digital Imaging and Communications (DICOM^1^) 3.0 standard creates a technical protocol for the storage and transmission of medical images and helps facilitate communication between multiple vendors and technologies in medicine.

In radiotherapy clinics, there are often circumstances which require modifying the properties of the DICOM images. As a commonly seen case in stereotactic radiosurgery, all MRI images (T1, T2, FLAIR) acquired within the same study will have the same frame of reference, or “Frame of Reference Unique Identifier (UID)”. This is a feature based on the understanding that the images are acquired in the same location. Unfortunately, this also means that any motion which occurs between scans cannot be corrected, as all images share the same Frame of Reference UID. To “break” this inherent registration, the DICOM value for Frame of Reference UID must be changed on each scan one wishes to register.

Changing any DICOM value often requires expert knowledge of file structure or specialized software, posing challenges and potential errors in accidentally or unnecessarily changing other attributes. This is especially challenging when a 4D‐CT is manipulated, as the Frame of Reference may need to be changed, but the new value must be consistent across the 4D scan. Barring these changes, the clinic would have to make do with imprecise registrations which add to overall treatment uncertainty.

Modifying DICOM properties is prone to error; attributes can be modified unintentionally, or files can be corrupted and hard to recover.^2^ Commonly used software can modify a subset of DICOM file attributes. For example, MIM^3^ allows the user to anonymize DICOM and change certain attributes, but this also rewrites many other DICOM attributes such as the date and time of creation. Raystation 10A^4^ has built‐in functionality to assign an exam to a new frame of reference, but this is the only DICOM attribute that can be changed and cannot be performed on examinations with an associated plan.

To address these gaps, we have created the Unlink program which provides a user‐friendly interface to change DICOM attributes which are not readily or easily available in commercial software. The simple interface offers the option to change three potential values: the Frame of Reference, Series Instance UID, and/or SOP Instance UID. The SOP Instance UID is defaulted to be always changed, however, users should note this will break associations with plan/structure sets.

Users can specify which modality they would like to change and use the built‐in unzip feature and run if files need to be extracted before being changed. This feature was added to facilitate an optimized workflow when pulling DICOM files from sites which are automatically zipped. This program, built in C#,^5^ is designed to run on any Windows based computer and is publicly available at https://github.com/BrianMAnderson/Unzip_Unlink_Csharp.

## 2 METHODS

The program was tested with publicly available brain MRI scans available here: https://figshare.com/articles/dataset/Data_from_An_Investigation_of_Machine_Learning_Methods_in_Delta‐radiomics_Feature_Analysis/9943334. This dataset contains several T1 and T2‐FLAIR images. Our program was then used to change the series instance UID, frame of reference UID, and SOP instance UID.

Verification of the edited DICOM files were evaluated within the RayStation 10A^4^ treatment planning system (TPS) and Varian's Eclipse TPS v16.1 (Varian Medical Systems, Palo Alto, CA). Further evaluation was performed with MIM^3^ to ensure that only the desired attributes were changed in the process.

## 3 SOFTWARE FORMAT AND USAGE NOTES

**FIGURE 1:**
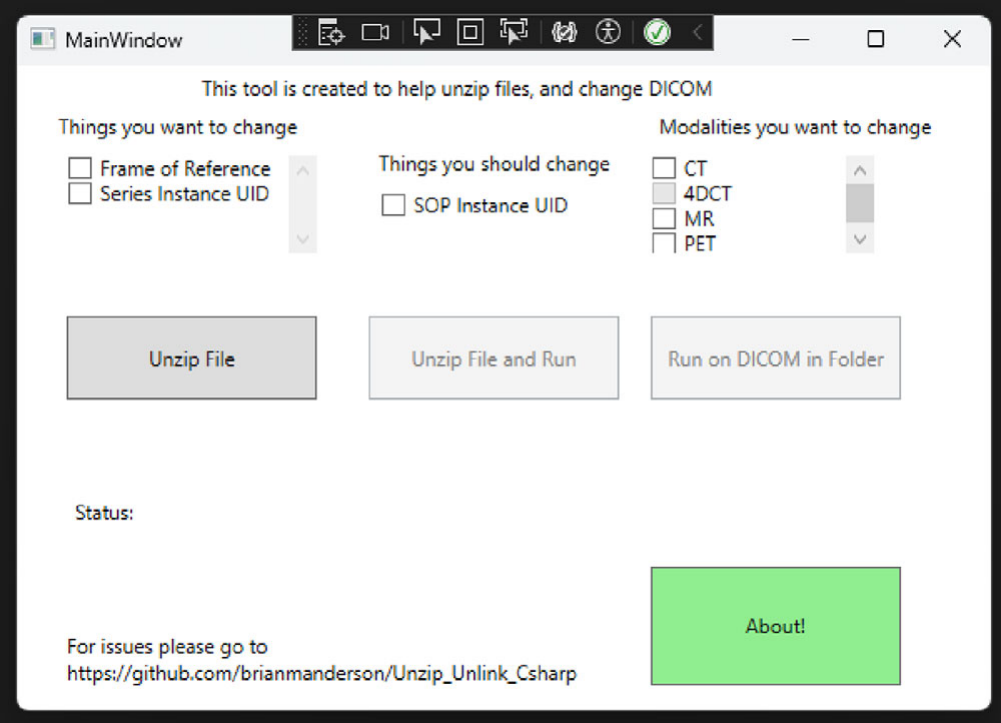
Main splash screen of the program. There are three check boxes of DICOM attributes that can be changed in the top left and middle, and three checkboxes for Modalities to change in the top right.

The program is written using C# and .NET framework 4.8, the current standard at time of creation (2023). All DICOM manipulation was facilitated with the FellowOak DICOM package ^6^ and SimpleITK.^7^ A demonstration of the program welcome screen is shown in Figure 1.

Users can select any or all the options in the upper left: Frame of Reference, Series Instance UID, and SOP Instance UID, as well as specify which modalities they would like to change: CT, 4DCT, MR, and/or PET images. The specification of modalities is beneficial when multiple modalities are located within the same folder.

Users may wish to change both the Frame of Reference UID and Series Instance UID if multiple registrations are required. For example, when registering a PET/CT to a new simulation scan, it can be useful to register both about the nasal cavity and also about the neck region. Two rigid registrations are sometimes not possible (within RayStation 10A^4^), and so having two distinct image sets which each has a unique registration can be advantageous to the physician during the delineation process.

New DICOM UIDs are generated using the FellowOak^4^ C# package.

### 3.1 4DCT registrations

An additional option for 4DCT was added because of the special nature of a 4DCT. Often, a free‐breathing scan *and* 4DCT are acquired at the same time. If the user wishes to change the frame of reference UID for the 4DCT, the program will need to create a unique frame of reference UID that is still consistent across all phases of the 4DCT, but distinct from the free‐breathing scan.

The program runs in two main steps. First, the program groups all files based on their unique Series Instance UIDs and modalities within the selected folder. Second, the DICOM files associated with each Series Instance UID are loaded, and for each selected attribute (Frame of Reference UID, Series instance UID, SOP instance UID) the associated tag is changed with the FellowOak^4^ package. After all changes have been applied, the new DICOM file is written over the original DICOM file.

When the 4DCT option is selected, any CT with the same frame of reference UID will be given a new frame of reference UID. This can be very useful when a 4DCT has the same frame of reference as a free‐breathing scan, and the user wishes to break this registration, but keep the 4DCT together.

### 3.2 Running the program

Note that DICOM files are required to be exported from the TPS and need to be in a folder accessible to the user. Once the DICOM attributes have been changed, the new data can be imported to the TPS.

**FIGURE 2:**
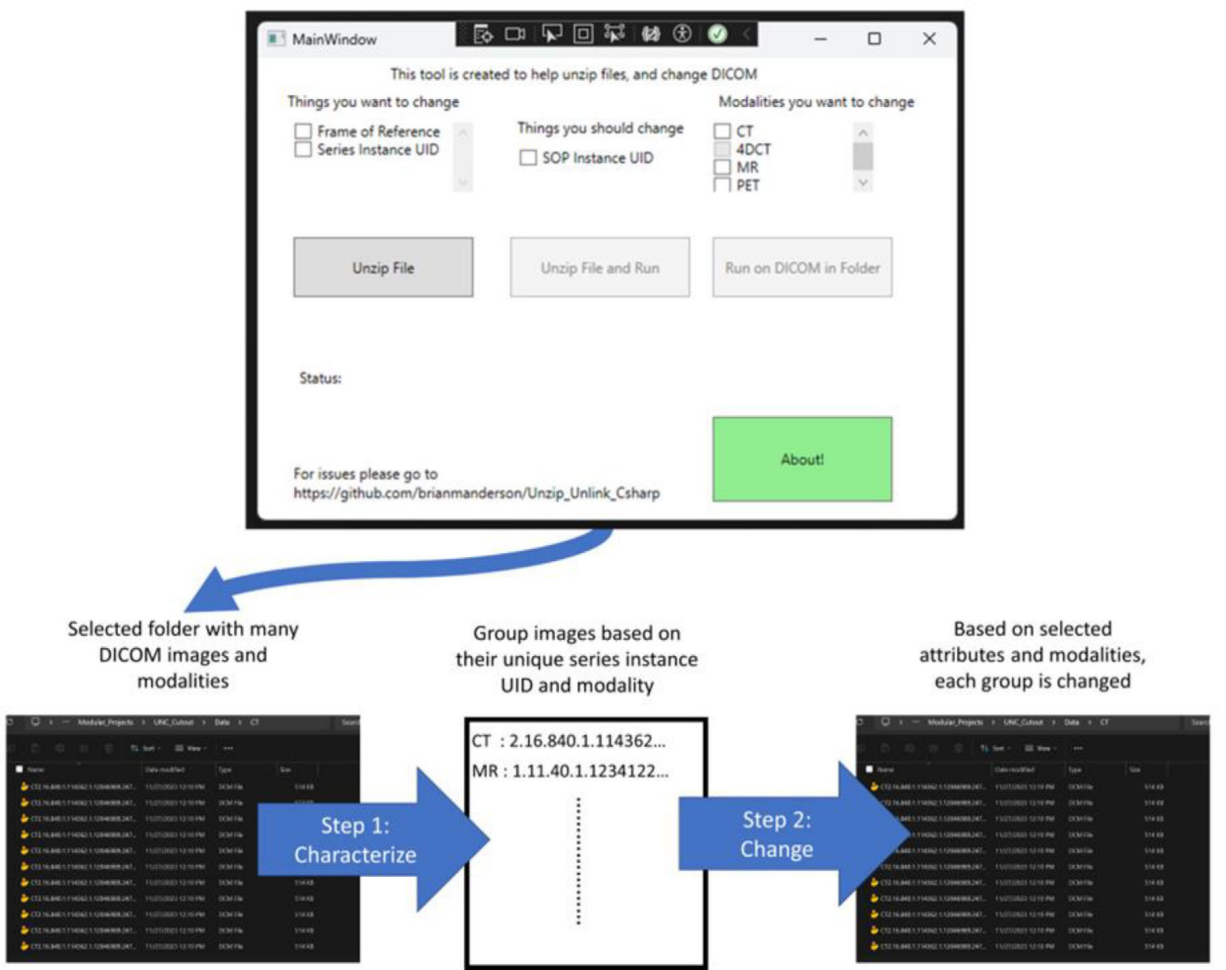
Graphical workflow of program.

A visual representation of the entire workflow can be seen in Figure 2. Green bars beneath the “Status” symbol give real‐time feedback of the updating process.

We noted that, depending on network speed, changing the attributes of a 125 slice CT scan required approximately 7 s. When the files are located on the local drive there is a significant increase in speed.

#### 3.2.1 Installation

The solution can be downloaded directly from GitHub with pre‐built executables or built directly from the source code.

## 4 RESULTS

Publicly available brain MRI images (https://figshare.com/articles/dataset/Data_from_An_Investigation_of_Machine_Learning_Methods_in_Delta‐radiomics_Feature_Analysis/9943334) and TCIA 4D CT Lung data^8^ were used as testing images to validate the software. The ability to change the frame of reference, series instance identifier, and SOP instance identifier using the program was evaluated with both the RayStation 10A^4^ TPS, MIM,^3^ and Varian's Eclipse v16.1. Within the TCIA 4DCT Lung data, changing attributes from the native Frame of Reference UID to a new UID that is still consistent across the 4DCT was also evaluated.

## 5 DISCUSSION

The program is designed to run on the Windows operating system and not macOS/Linux. There is concern that internet security requirements may prevent the installation of this program onto a computer. Within our institution we were able to circumnavigate this issue by placing the compiled program on a network drive location which was accessible to the team, which runs without the requirement of installation.

### 5.1 Potential applications

The program presented here represents an easy, user‐friendly method of changing two commonly changed DICOM attributes with a vendor agnostic solution. This can be useful when breaking the inherent registration of consecutively acquired MRI images, registering a free breathing scan to a breath hold scan, or a free breathing scan to a 4D CT. Because we have hosted the tool on GitHub, any user can provide feedback and new attributes can easily be added to the program in the future.

We have implemented this solution within two clinics: University of North Carolina at Chapel Hill and University of California, San Diego with positive feedback from the physics and dosimetry teams. The program is freely available and open for input from the community via GitHub, allowing future updates and improvements as requested.

## CONFLICT OF INTEREST STATEMENT

The authors declare no conflicts of interest.

## FUNDING INFORMATION

None

## Data Availability

Research data are available at https://github.com/brianmanderson/Unzip_Unlink_Csharp. Image data available at https://figshare.com/articles/dataset/Data_from_An_Investigation_of_Machine_Learning_Methods_in_Delta‐radiomics_Feature_Analysis/9943334, https://www.cancerimagingarchive.net/collection/4d‐lung/.
